# Effect of ethnicity and other sociodemographic factors on attendance at ophthalmology appointments following referral from a Diabetic Eye Screening Programme: a retrospective cohort study

**DOI:** 10.1136/bmjophth-2024-001969

**Published:** 2025-01-22

**Authors:** James Banks, Abraham Olvera-Barrios, Matilda Pitt, Daisy Williams, Michael Seltene, Celestine Rutowska, Mumina Khatun, Josef Huemer, Yasir Khan, Zoe Ockrim, Ling Zhi Heng, Alicja R Rudnicka, Adnan Tufail, Catherine A Egan, Christopher G Owen

**Affiliations:** 1 Population Health Research Institute, City St George's, University of London , London, UK; 2 Medical Retina, Moorfields Eye Hospital NHS Foundation Trust, London, UK; 3 NIHR Biomedical Research Centre at Moorfields Eye Hospital NHS Foundation Trust and UCL Institute of Ophthalmology, London, UK

**Keywords:** Retina, Epidemiology, Macula, Public health

## Abstract

**Background/aims:**

To examine the association between sociodemographic characteristics and attendance at Hospital Eye Service (HES) referrals from the Diabetic Eye Screening Programme (DESP), in a large, ethnically diverse urban population.

**Methods:**

Retrospective cohort study (4 January 2016–12 August 2019) of people with diabetic retinopathy (DR) referred from an English DESP to a tertiary referral eye hospital. We conducted a multivariable logistic regression with attendance as the primary outcome, controlling for age, sex, ethnicity, Index of Multiple Deprivation, best eye visual acuity and baseline DR grade.

**Results:**

Of 7793 people referred (mean age 64 years, 62.6% male, 13.9% white, 12.5% black, 25.3% South Asian, 6.5% any other Asian background, 19.3% no recorded ethnicity and 20.9% of ‘Other’ ethnic origin), 69% attended. Compared with white individuals, people of black ethnic origin were similarly likely to attend. South Asians and those of other Asian backgrounds were more likely, and people with ‘Other’ or missing ethnicity were less likely to attend. Those with higher levels of deprivation, younger (aged 18–45 years) and older (76–90 years) age groups and worse visual acuity were less likely to attend, whereas people identified as having proliferative DR in both eyes were more likely to attend.

**Conclusion:**

Sociodemographic patterns in attendance after referral from the DESP to the HES exist, and these do not appear to explain ethnic differences in more severe sight-threatening DR, suggesting other explanations. More work is needed to understand and reduce inequalities in HES attendance.

**WHAT IS ALREADY KNOWN ON THIS TOPIC**

- Sociodemographic factors including ethnicity are associated with low attendance at the Diabetic Eye Screening Programme (DESP), but it is unclear how these factors influence attendance at Hospital Eye Service (HES) referrals.

**WHAT THIS STUDY ADDS**

- This is the first study to show at scale that sociodemographic factors other than ethnicity are more strongly associated with low attendance at referrals from the DESP to the HES in a UK context.

**HOW THIS STUDY MIGHT AFFECT RESEARCH, PRACTICE OR POLICY**

- We suggest measures that might reduce inequalities in attendance at DESP referrals to the HES.

## Introduction

 Diabetic retinopathy (DR) is a common cause of sight loss worldwide and a major cause of blindness in the UK working-age population.^1^,^3^ Early detection and timely treatment are essential to prevent sight loss.^4^ The NHS Diabetic Eye Screening Programme (DESP) is effective in identifying patients with possible sight-threatening diabetic retinopathy (STDR) and referring them to the Hospital Eye Service (HES) for further ophthalmological management.^5^ However, non-attendance at HES appointments prevents patients from accessing timely treatment, increasing the risk of irreversible visual impairment and blindness. While there have been previous studies which explored how ethnicity and sociodemographic factors affect UK DESP attendance^6^,^13^ and work looking at factors affecting adherence to postophthalmic screening tertiary referrals in a Singaporean context,^14^ how these factors affect attendance at HES Medical Retina appointments in the UK among those referred from the DESP with STDR is less well understood, and as far as we know has not been examined at scale.

London is one of the most sociodemographically diverse areas in the UK, with marked disparities in deprivation at a small area level.^15^ The four authorities responsible for developing plans^16^,^19^ to meet the health needs of local populations in London have all recognised health inequalities among the Core20 population, identified as the most deprived fifth of the population nationally,^20^ as a key priority. Diabetes care is high on the Core20PLUS5 agenda and is a specific priority in the approach put forward, especially among younger people.^21^ Among people living with diabetes, this study examines sociodemographic factors affecting attendance at referrals to HES Medical Retina appointments from the regional South West London (SWL) DESP, in this large, ethnically diverse population with wide-ranging levels of deprivation.

## Methods

This was a retrospective cohort study examining attendance at HES Medical Retina appointments occurring between January 2016 and August 2019. The determinants of attendance available for study were age, sex, self-reported ethnicity, area-level deprivation (Index of Multiple Deprivation (IMD^22^)), visual acuity and DR grade following the UK National Screening Committee classification system.^23^

### Setting

The UK DESP offers regular diabetic eye screening to all people with diabetes aged 12 years or older. People were offered multiple opportunities to attend a Routine Digital Screening appointment, at which their visual acuity was assessed and two 45° digital retinal images centred on the fovea and disc were taken of each eye. Patients with maculopathy (M1), preproliferative (R2) or proliferative DR (R3) are referred to HES. Moorfields Eye Hospital is one of the largest tertiary referral centres in England and serves five main DESPs (ie, North Central London, North East London, North West London, South East London and South West London DESP). Any person who attended a Medical Retina ophthalmology appointment during the study period was defined as ‘attended’. Only those who failed to attend all appointments offered in the period and were subsequently discharged were classified as ‘Did not attend’.

### Data extraction

Anonymised data were extracted using a structured query language from the health records of all people aged 18 years or more who were referred from the DESP for a Medical Retina appointment during the study period.

### Independent variable recording

#### Ethnicity

Self-reported ethnicity data were obtained either from patients at their screening appointment or from ethnicity data provided by their general practitioner surgery and recorded in the nationally mandated screening software. Ethnicity data for those who attended were categorised according to the five census high-level ethnic groups (defined as Asian, black, mixed, white and other ethnic groups).^24^ Ethnicity data pertaining to the 19 ethnic subcategories were available from DESP data for those who did not attend HES referrals, and these were sorted into the high-level ethnic categories to allow comparison between datasets.

#### Index of Multiple Deprivation

In order to measure relative deprivation in England, each area or neighbourhood of approximately 1500 inhabitants (a lower-layer super output area (LSOA)) is ranked from 1st, the most deprived, to 32, 844th, the least deprived, according to 39 indicators in seven different domains. Patients’ postcodes were used to identify the IMD score of their LSOA.

#### Visual acuity

Baseline visual acuity was recorded using Snellen, logMAR or ETDRS (Early Treatment Diabetic Retinopathy Study) notation and converted to Snellen notation to enable comparison. Baseline visual acuity was defined as the best measurement obtained from the first HES visit or, from the referral made by the DESP, if the patient did not attend. The best-seeing eye visual acuity score was assigned to each person.

#### Maculopathy and retinopathy

Maculopathy and retinopathy grades were recorded, either from the first HES visit or from the referral made by the DESP, and DR codes were simplified into non-proliferative STDR in one eye (R2 or M1 in one eye), non-proliferative STDR in both eyes, proliferative DR (R3, PDR) in one eye and PDR in both eyes.

#### Statistical analysis

Multivariable logistic regression analysis of attendance at a hospital retina appointment was performed (binary outcome coded ‘1’ if patient attended and ‘0’ if they did not attend). Independent variables considered were age, sex, ethnicity, IMD, visual acuity (best eye, with a secondary analysis examining worst eye) and DR grade. Age and visual acuity were categorised (table 1) to accommodate non-linear patterns in attendance. Rank scores of the IMD were divided into quintiles with the first quintile being the most deprived and the fifth quintile the least deprived. R V.4.3.1 was used for statistical analysis.^25^

**Table 1:** Summary table of patient characteristics

Characteristic	Number (%)
Age groups in years	
18–30	102 (1.3%)
31–45	645 (8.3%)
46–60	2386 (30.6%)
61–75	3070 (39.4%)
76–90	1463 (18.8%)
>90	127 (1.6%)
Mean age (SD) years	64.1 (13.84)
Sex	
Male	4875 (62.6%)
Female	2918 (37.4%)
Ethnicity	
White British	1087 (13.9%)
Mixed	92 (1.2%)
Black	972 (12.5%)
South Asian	1974 (25.3%)
Chinese	24 (0.3%)
Any other Asian background	509 (6.5%)
Other	1629 (20.9%)
Missing	1506 (19.3%)
Index of Multiple Deprivation (IMD) categories	
1 (most deprived)	1592 (20.4%)
2	2903 (37.3%)
3	1818 (23.3%)
4	1026 (13.2%)
5 (least deprived)	454 (5.8%)
Better eye visual acuity (Snellen)	
Better than 6/6 best eye	7005 (89.9%)
6/6 to 6/9 best eye	544 (7%)
6/9 to 6/18 best eye	160 (2.1%)
<6/18 best eye	84 (1.1%)
Better than 6/6 worst eye	5919 (76.0%)
6/6 to 6/9 worst eye	955 (12.3%)
6/9 to 6/18 worst eye	460 (5.9%)
<6/18 worst eye	459 (5.8%)
Level of diabetic retinopathy	
Non-proliferative STDR in one eye (R2 or M1 in one eye)	2904 (37.3%)
Non-proliferative STDR in both eyes	3232 (41.5%)
PDR in one eye	543 (7%)
PDR in both eyes	1114 (14.3%)
Total	7793 (100%)

IMDIndex of Multiple DeprivationPDRProliferative Diabetic RetinopathySTDRSight Threatening Diabetic Retinopathy

#### Patient and public involvement

We plan to disseminate the findings of our study to people eligible for diabetic eye screening and their families through the local press and via social media. In addition, we intend to seek wider dissemination to the public through the English national screening programme’s communication team.

## Results

A total of 12 253 DESP patient referrals to Moorfields Eye Hospital Medical Retina Service were identified during the study period. Of these, 7793 were included in the analysis (4431 patients excluded due to non-DR referrals, and 29 patients with missing data on age, sex, IMD and visual acuity). Table 1 summarises the characteristics of participants. Mean age was 64.1 years (SD 13.8), and 62.6% were male. 81% of the cohort had usable ethnicity records. The majority were South Asian (25.3%), followed by those whose ethnicity was coded as Other (20.9%). A majority 57.7% lived in areas with the highest levels of deprivation (defined as the first to second IMD quintiles). Overall, appointment attendance during the study period was 69%.

ORs of attendance at HES Medical Retina referrals by patient characteristics are given in table 2. Up to the age of 75 years, increasing age was associated with better attendance, with those aged 61–75 most likely to attend (OR=1.2; 95% CI 1.1 to 1.4, p=0.001, with adjustment) compared with the 46–60 year old reference group. After the age of 75 years, individuals were increasingly less likely to attend. Those aged 18–45 years showed poorer attendance when compared with the reference group. After adjustment, participants aged 18–30 years were least likely to attend their HES appointment (OR=0.5; 95% CI 0.3 to 0.8, p=0.002).

**Table 2:** ORs of attendance at Hospital Eye Service Medical Retina appointments from the diabetic eye screening programme by patient characteristics

Characteristic	Attended	DNA	Univariable (CI, p value)	Multivariable adjusted* (CI, p value)
Age (years)				
18–30	53	49	0.53 (0.36 to 0.79, **0.002**)	0.51 (0.34 to 0.78, **0.002**)
31–45	387	258	0.74 (0.62 to 0.88, **0.001**)	0.67 (0.56 to 0.81,<**0.001**)
46–60 (Reference)	1600	786	1	1
61–75	2276	794	1.41 (1.25 to 1.58,<**0.001**)	1.24 (1.10 to 1.40, **0.001**)
76–90	988	475	1.02 (0.89 to 1.17, 0.761)	0.81 (0.70 to 0.95, **0.007**)
>90	70	57	0.60 (0.42 to 0.86, **0.006**)	0.54 (0.37 to 0.79, **0.002**)
Sex				
Male	3332	1543	1	1
Female	2042	876	1.08 (0.98 to 1.19, 0.132)	1.10 (0.99 to 1.22, 0.069)
Ethnicity				
White British (Reference)	769	318	1	1
Mixed	66	26	1.05 (0.65 to 1.68, 0.840)	1.10 (0.68 to 1.78, 0.694)
Black	688	284	1.00 (0.83 to 1.21, 0.985)	1.07 (0.88 to 1.30, 0.495)
South Asian	1560	414	1.56 (1.31 to 1.85,<**0.001**)	1.50 (1.26 to 1.78,<**0.001**)
Chinese	18	6	1.24 (0.49 to 3.15, 0.651)	1.11 (0.43 to 2.86, 0.835)
Any other Asian background	403	106	1.57 (1.22 to 2.02,<**0.001**)	1.47 (1.14 to 1.90, **0.003**)
Any other ethnic group	1080	549	0.81 (0.69 to 0.96, **0.015**)	0.80 (0.67 to 0.95, **0.01**)
Missing	790	716	0.46 (0.39 to 0.54,<**0.001**)	0.45 (0.38 to 0.54,<**0.001**)
Index of Multiple Deprivation				
1 (Reference)—most deprived	1076	516	1	1
2	1978	925	1.03 (0.90 to 1.17, 0.706)	1.05 (0.92 to 1.21, 0.458)
3	1275	543	1.13 (0.97 to 1.30, 0.109)	1.33 (1.14 to 1.56,<**0.001**)
4	707	319	1.06 (0.90 to 1.26, 0.479)	1.30 (1.09 to 1.56, **0.004**)
5—least deprived	338	116	1.40 (1.10 to 1.77, **0.005**)	1.79 (1.39 to 2.30, **<0.001**)
Better eye visual acuity				
Better than 6/6	4806	22 199	0.83 (0.68 to 1.01, 0.064)	1.02 (0.83 to 1.26, 0.846)
6/6 to 6/9 (Reference)	394	150	1	1
6/9 to 6/18	122	38	1.22 (0.81 to 1.84, 0.337)	1.13 (0.73 to 1.73, 0.585)
<6/18	52	32	0.62 (0.38 to 1.00, **0.049**)	0.53 (0.32 to 0.87, **0.013**)
Level of diabetic retinopathy				
Non-proliferative STDR in one eye (R2 or M1 in one eye) (reference)	1862	1042	1	1
Non-proliferative STDR in both eyes	2183	1049	1.16 (1.05 to 1.29, **0.005**)	1.16 (1.04 to 1.29, **0.01**)
PDR in one eye	382	161	1.33 (1.09 to 1.62, **0.005**)	1.28 (1.04 to 1.57, **0.02**)
PDR in both eyes	947	167	3.17 (2.65 to 3.80,<**0.001**)	3.03 (2.51 to 3.67,<**0.001**)

* Mutually adjusted for age, sex, ethnicity, IMD, Vvisual acuity and diabetic retinopathy.

DNA, did not attend; PDR, proliferative diabetic retinopathySTDR, sight threatening diabetic retinopathy

Compared with white individuals, those of South Asian ethnicity (OR=1.5 95% CI 1.3 to 1.8, p<0.001) and any other Asian background (OR=1.5; 95% CI 1.1 to 1.9, p=0.003) showed increased odds of attendance after adjustment. However, odds of attendance were lower among individuals with missing ethnicity data (OR=0.5; 95% CI 0.4 to 0.5, p<0.001) and with ethnicities recorded as ‘Any other ethnic group’ (OR=0.8; 95% CI 0.7 to 1.0, p=0.01) when compared with white individuals, after adjustment. A further analysis (online supplemental table 2) of percentage attendance by ethnicity and sex did not reveal further significant patterns.

Adjusted analyses showed that individuals living in more deprived areas (first and second IMD quintiles) were less likely to attend their screening appointments (linear trend for IMD, adjusted for all covariates=1.138 (1.086, 1.192, p<0.001)). Those in the least deprived fifth IMD quintile were much more likely to attend than those in the most deprived first IMD quintile (OR=1.8; 95% CI 1.4 to 2.3, p<0.001).

Odds of attendance for those with visual acuity worse than 6/18 were significantly decreased (OR=0.5; 95% CI 0.3 to 0.9, p=0.013). Note that this pattern of association was not apparent using worst eye visual acuity (online supplemental table 1), only that acuity better than 6/6 in the worst eye (ie, good acuity in both eyes) was associated with less attendance. Furthermore, while there was an increased odds of attendance among those with non-proliferative STDR in both eyes (OR=1.2, 95% CI 1.0 to 1.3, p=0.01) and PDR in one eye (OR=1.3; 95% CI 1.04 to 1.29, p=0.02), the most striking increase in odds of attendance was for those with PDR in both eyes (OR=3.0, 95% CI 2.5 to 3.7, p<0.001).

## Discussion

This study shows marked sociodemographic differences in attendance at referrals from five regional DESPs to HES appointments among people living with diabetes at high risk of sight loss from DR. We show that the youngest and oldest age groups were less likely to attend referrals compared with those 46–60 years of age. South Asian patients and those of other Asian backgrounds were more likely and those of mixed or with missing ethnicity data were less likely to attend compared with those of white ethnicity. Importantly, in a universal healthcare setting where healthcare delivery is limited by system capacity rather than by patient’s economic circumstances, such as the NHS, we show a strong relationship between levels of deprivation and attendance, with those in the least deprived three quintiles more likely to attend than those in the most deprived areas. Reassuringly, those at the highest risk of sight loss (ie, with PDR in both eyes) and in most need of ophthalmological assessment were three times more likely to attend. Services already place a particular emphasis on trying to engage with those at the highest risk of sight loss (R3), but more work is required into initiatives which may further improve appointment uptake, for example, walk-in clinics direct from screening.

Previous studies have shown increased rates of DR and more advanced STDR in both black and Asian populations when compared with white people.^11 26 27^ However, we and others have shown that this cannot be simply explained by ethnic differences in DESP attendance.^2 11 13^ Previous work has shown that delays in referral from DESP to the HES among those with more advanced DR, that is, PDR, can result in visual loss.^28^ Hence, we sought to examine whether there are sociodemographic differences in attendance at referrals from the DESP to the HES among those at high risk of sight loss, which could potentially account for ethnic disparities in STDR. Our findings suggest that the higher rates of STDR among South Asian and black ethnic groups cannot simply be explained by decreased attendance at HES (Medical Retina) referral appointments. Other mechanisms related to ethnicity, such as susceptibility and/or diabetic control, require further exploration.^29^ Another important group to consider are those with missing ethnicity data, which was appreciable in our data set (19.3%). Poor ethnicity data recording limits research into sociodemographic determinants of health,^30^ and improvement in ethnicity recording may lead to a greater understanding of barriers to attending Medical Retina appointments for those with missing ethnicity data. Moreover, better characterisation of those broadly categorised as ‘Any other ethnic background’ who were also less likely to attend needs to be unpacked further to elucidate reasons for non-attendance. A task force to tackle the issue of missing ethnicity data has shown that this can lead to dramatic improvements in the rate of recording, providing a mechanism to improve data quality in future.^31^ Future initiatives should take into account recent work into the barriers for professionals and patients when it comes to talking about ethnicity, including patient concerns regarding potential discrimination, and professional lack of confidence and comfortableness about asking.^32^

Odds of attendance were decreased for both the oldest (>75 years) and youngest (<46 years) individuals. In line with previous studies of DESP attendance, patients aged 18–45 were less likely to attend an appointment than those aged 45–60. Prothero *et al*^33^ suggested that anxiety about DR, lack of appointment flexibility, difficulties in obtaining time off from work and study commitments, and a lack of integration of diabetic eye screening appointments with other diabetes appointments are key barriers and that younger people would benefit from a more tailored approach. It is highly likely that similar factors influence attendance once referred to the HES with more severe STDR.

Our findings also concur with earlier studies that have shown that socioeconomic deprivation is closely linked to attendance at eyecare appointments for people with diabetes.^2 12 13^ The most deprived 20% were least likely to attend referral to the HES, and this group represents the Core20 population at the heart of NHS England’s current focus on reducing health inequalities as part of the Core20PLUS5 initiative.^16 17^ Given the multiple components which comprise IMD (income, employment, education, health, crime, barriers to housing and services and living environment), it is challenging to identify ways in which providers of eyecare to patients with diabetes can reduce inequalities for those who use their services. However, it is known that poor-quality housing harms physical and mental health in many ways.^34^ In the context of healthcare appointment attendance, unstable housing has been shown to be related to suboptimal clinic attendance.^35^ In the absence of stable addresses, professionals are reliant on mobile phone numbers to reach patients. However, studies have shown that patients changing their mobile phone numbers also results in patients being lost to follow-up, and that loss and theft of mobile phones can severely limit their effectiveness as a method of arranging follow-up in some patients.^36 37^ A recent rapid review by Davey *et al*^38^ suggests five principles for ‘levelling up’ health, and their emphasis on interventions with a locally designed focus and interventions targeted at disadvantaged communities may be of particular relevance to those organising local eyecare services for people with diabetes. In SWL, a focus on the borough containing 50% of Core20 residents (ie, Croydon) would be particularly worthwhile. Offering appointments on days and at times that do not disadvantage people on zero-hours contracts or with caring responsibilities might be a practical step worth exploring in an area with low average incomes and high levels of employment instability.

The association of worse visual acuity with non-attendance replicates the findings of our previous study looking at attendance in a large, multiethnic DESP in North East London.^13^ However, this study is novel in examining referrals from the DESP to the HES and in showing that those with good acuity in both eyes have lower odds of attendance. Future studies examining referrals to the HES with more advanced DR would benefit from the inclusion of data relating to duration and type of diabetes in order to further contextualise this association. While it is true that lower attendance among those with poor vision could be attributed to other non-DR causes, non-DR referrals were excluded from the analyses. Future studies could include more data pertaining to participants’ wider ophthalmic health. The association of proliferative STDR with increased odds of attendance at Medical Retina HES appointments is a reassuring finding as these patients are most in need of treatment. It is likely that the identification of diabetic eye complications heralding the possibility of future blindness serves as a significant motivating factor for people living with diabetes to attend such appointments. As such, the delivery of this news at or following a retinopathy screening programme appointment may constitute a ‘teachable moment’ which could serve as an opportunity to motivate patients with diabetes to adopt risk-reducing health behaviours.^39^

Our study has several strengths. First, it is to our knowledge the first study to look at the sociodemographic factors affecting attendance at scale and at this point of the care pathway for people with STDR in the UK, and consequently the first study to demonstrate similarities in determinants of non-attendance at diabetic eye screening and referrals to medical retina, thus establishing a pattern of behaviour irrespective of disease severity. Second, we examine the association between the grade of retinopathy and attendance at specialist retinal clinic appointments referred from the DESP, which we also believe to be a novel approach. Third, the study was carried out using data from one of the main referral centres for ophthalmic diseases in England serving London-based DESPs covering an area with high levels of sociodemographic diversity, with patients referred from DESPs which are quality assured and conform to national recommendations. Lastly, the large sample size allowed for sufficient power to detect associations between attendance at appointments and age, sex, IMD quintile, visual acuity and grade of retinopathy which would not have been possible in a smaller study. There are some drawbacks. First, missing ethnicity data prevent a full understanding of the characteristics of a disenfranchised group, although we have usable ethnicity data for over 80% of our cohort. Second, the absence of data relating to traditional risk factors for DR, that is, metabolic data, were not available. Third, data on the number of appointments offered and not attended for those eventually discharged were incomplete. Fourth, there are numerous reasons for non-attendance (many of which we examine); this could include that they received tertiary care from elsewhere, especially in London where different specialist centres exist. Unfortunately, we do not have the data to examine this.

Further work is required to identify evidence-based strategies to tackle health inequalities in attending referrals from the DESP to the HES among people living with diabetes at high risk of visual loss, given the mounting evidence that increased deprivation is strongly and pervasively associated with decreased attendance.

## Conclusion

In a large, multiethnic population, non-white ethnicity is not associated with poor attendance at HES referrals. Social deprivation, age and visual acuity were shown to be more strongly associated with low attendance. In England, inequalities impacting diabetic eye care are directly relevant to the current Core20PLUS5 agenda, and these findings provide further insight into the potential facilitators and barriers of attendance.

## supplementary material

10.1136/bmjophth-2024-001969 online supplemental table 1

## Data Availability

All data relevant to the study are included in the article or uploaded as supplementary information.
