# Supplementary material for: Effect of ethnicity and other sociodemographic factors on attendance at ophthalmology appointments following referral from a Diabetic Eye Screening Programme: a retrospective cohort study
Source: BMJ Open Ophthalmol. 2025 Jan 22;10(1):e001969. doi: 10.1136/bmjophth-2024-001969 (PMC11759212; doi:10.1136/bmjophth-2024-001969)
Supplement: online supplemental table 1 [file bmjophth-10-1-s001.pdf]

Supplemental Table 1: Odds ratios of attendance at Hospital Eye Service Medical Retina appointments from the diabetic eye screening programme by patient characteristics using worst eye visual acuity

Characteristic	Attended	DNA	Univariate (CI, p-value)	Multivariate Adjusted* (CI, p-value)
**Age**				
18-30	53	49	0.53 (0.36-0.79, **0.002**)	0.53 (0.35-0.8, **0.003**)
31-45	387	258	0.74 (0.62-0.88, **0.001**)	0.69 (0.57-0.83, **<0.001**)
46-60 (Reference)	1600	786	1	1
61-75	2276	794	1.41 (1.25-1.58, **<0.001**)	1.19 (1.05-1.34, 0.007)
76-90	988	475	1.02 (0.89-1.17, 0.761)	0.71 (0.61-0.83, **<0.001**)
>90	70	57	0.60 (0.42-0.86, **0.006**)	0.44 (0.30-0.64, **<0.001**)
**Sex**				
Male	3332	1543	1	1
Female	2042	876	1.08 (0.98-1.19, 0.132)	1.09 (0.98-1.21, 0.124)
**Ethnicity**				
White British (Reference)	769	318	1	1
Mixed	66	26	1.05 (0.65-1.68, 0.84)	1.10 (0.68-1.79, 0.685)
Black	688	284	1.00 (0.83-1.21, 0.985)	1.06 (0.87-1.29, 0.555)
South Asian	1560	414	1.56 (1.31-1.85, **<0.001**)	1.49 (1.25-1.77, **<0.001**)
Chinese	18	6	1.24 (0.49-3.15, 0.651)	1.09 (0.42-2.82, 0.857)
Any other Asian background	403	106	1.57 (1.22-2.02, **<0.001**)	1.48 (1.14-1.91, **0.003**)
Other	1080	549	0.81 (0.69-0.96, **0.015**)	0.81 (0.68-0.96, **0.015**)
Missing	790	716	0.46 (0.39-0.54, **<0.001**)	0.46 (0.38-0.54, **<0.001**)
**Index of Multiple Deprivation**				
IMD:1 (Reference)	1076	516	1	1
IMD:2	1978	925	1.03 (0.9-1.17, 0.706)	1.07 (0.93-1.22, 0.365)
IMD:3	1275	543	1.13 (0.97-1.3, 0.109)	1.37 (1.18-1.61, **<0.001**)
IMD:4	707	319	1.06 (0.9-1.26, 0.479)	1.34 (1.12-1.61, **0.001**)
IMD:5	338	116	1.40 (1.1-1.77, **0.005**)	1.88 (1.47-2.42, **<0.001**)
**Worst eye visual acuity**				
VA: Better than 6/6	3914	2005	0.62 (0.53-0.73, **<0.001**)	0.69 (0.59-0.82, **<0.001**)
VA: 6/6 to 6/9 (Reference)	725	230	1	1
VA: 6/9 to 6/18	368	92	1.27 (0.97-1.67, 0.087)	1.13 (0.85-1.49, 0.415)
VA: <6/18	367	92	1.27 (0.96-1.66, 0.09)	1.20 (0.9-1.59, 0.219)
**Level of diabetic retinopathy**				
Non - proliferative STDR in one eye (R2 or M1 in one eye) (Reference)	1862	1042	1	1
Non - proliferative STDR in both eyes	2183	1049	1.16 (1.05-1.29, **0.005**)	1.15 (1.03-1.29, **0.012**)
PDR in one eye	382	161	1.33 (1.09-1.62, **0.005**)	1.14 (0.93-1.4, 0.219)
PDR in both eyes	947	167	3.17 (2.65-3.8, **<0.001**)	2.61 (2.16-3.16, **<0.001**)

*Mutually adjusted for age, sex, ethnicity, IMD, Visual acuity and diabetic retinopathy

Supplemental Table 2: Percentage attending Hospital Eye Service Medical Retina appointments from the diabetic eye screening programme by ethnicity and sex

	Male (95% CI)	Female (95% CI)
**White British**	70.2% (66.7, 73.7)	71.6% (67.4, 75.9)
**Mixed**	71.4% (58.8, 84.1)	72.1% (58.7, 85.5)
**Black**	68.8% (64.9, 72.8)	73.2% (69.0, 77.3)
**South Asian**	80.2% (77.9, 82.4)	77.3% (74.3, 80.2)
**Chinese**	69.2% (44.1, 94.3)	81.8% (59, 100)
**Any other Asian background**	79.3% (75.2, 83.5)	78.7% (72.0, 85.5)
**Other**	64.8% (62.0, 67.7)	69% (65.2, 72.8)
**Missing**	52.3% (49.3, 55.4)	52.7% (48.3, 57.0)
